# Pofatu, a curated and open-access database for geochemical sourcing of archaeological materials

**DOI:** 10.1038/s41597-020-0485-8

**Published:** 2020-05-11

**Authors:** Aymeric Hermann, Robert Forkel, Andrew McAlister, Arden Cruickshank, Mark Golitko, Brendan Kneebone, Mark McCoy, Christian Reepmeyer, Peter Sheppard, John Sinton, Marshall Weisler

**Affiliations:** 1 Department of Linguistic and Cultural Evolution, Max Planck Institute for the Science of Human History, 07745 Jena, Thüringen Germany; 2 CNRS, UMR 7041 ArScAn, Equipe Ethnologie Préhistorique, Maison de l’Archéologie et de l’Ethnologie, CNRS, UMR 7041, 92023 Nanterre, France; 3 Department of Anthropology, School of Social Sciences, University of Auckland, Auckland, 1010 New Zealand; 4 CFG Heritage Ltd, Auckland, 1010 New Zealand; 5 Department of Anthropology, University of Notre Dame, Indiana, 46556 USA; 6 Department of Anthropology, Southern Methodist University, Dallas, Texas 75205 USA; 7 College of Arts, Society and Education, James Cook University, Cairns, Queensland 4878 Australia; 8 Department of Geology and Geophysics, University of Hawai’i at Mānoa, Hawai’i, 96822 USA; 9 School of Social Science, University of Queensland, St Lucia, Queensland 4072 Australia

**Keywords:** Geochemistry, Archaeology

## Abstract

Compositional analyses have long been used to determine the geological sources of artefacts. Geochemical “fingerprinting” of artefacts and sources is the most effective way to reconstruct strategies of raw material and artefact procurement, exchange or interaction systems, and mobility patterns during prehistory. The efficacy and popularity of geochemical sourcing has led to many projects using various analytical techniques to produce independent datasets. In order to facilitate access to this growing body of data and to promote comparability and reproducibility in provenance studies, we designed *Pofatu*, the first online and open-access database to present geochemical compositions and contextual information for archaeological sources and artefacts in a form that can be readily accessed by the scientific community. This relational database currently contains 7759 individual samples from archaeological sites and geological sources across the Pacific Islands. Each sample is comprehensively documented and includes elemental and isotopic compositions, detailed archaeological provenance, and supporting analytical metadata, such as sampling processes, analytical procedures, and quality control.

## Abstract (summary)

**Table:** 

Measurement(s)	isotopic composition • chemical composition • contextual information for archaeological sources • contextual information for stone artefacts
Technology Type(s)	digital curation • computational modeling technique
Factor Type(s)	archaeological provenance • artefact attribute
Sample Characteristic - Location	Region (Papua New Guinea) • Vanuatu Islands • Solomon Islands • Fiji islands • Tonga Archipelago • Samoa • American Samoa • Wallis and Futuna Islands • Tuvalu Islands • Tokelau Islands • Rotuma Island Group • Cook Islands • Society Islands • Marquesas Islands • Archipel des Tuamotu • Gambier Islands • Austral Islands • Pitcairn, Henderson, Ducie and Oeno Islands • Easter Island • State of Hawaii • Micronesia • New Zealand

Machine-accessible metadata file describing the reported data: 10.6084/m9.figshare.12073116

## Background & Summary

Extracting, transforming, and distributing natural resources and finished goods between individuals and groups has always been an important aspect of technological, economic, and social behaviors in human societies^1–4^. Such material aspects of cultures can be inferred with the help of provenance studies, by reconstructing the movements of materials and artefacts across space. For this purpose, archaeologists have regularly used petrographic and geochemical analyses for more than 40 years for characterising the geological provenance of raw materials and stone artefacts and for reconstructing patterns of exchange based on hard evidence^5–7^. Geochemical techniques have proven to be the most efficient and reliable way to fingerprint raw material sources and artefacts thereby providing reproducible and comparable results^8–10^. Furthermore, geochemical data are quantitative and can therefore be examined with statistical methods^11,12^ or by using, for example, well-known principles of petrogenesis and mantle source evolution.

Due to the improvement of analytical techniques and the increasing use of geochemical sourcing, the production and publication of archaeological compositional data have grown exponentially. It is now recognized that using large source data compilations can lead to more efficient and cost-effective research planning^7,10,13^. Sharing source data compilations facilitates assigning unambiguous provenance to artefacts because it enables a better understanding of geochemical variability of sources throughout a given study region and also shows potential geochemical differences between sources^14^, especially for artefacts found in either very homogeneous or complex petrogenetic contexts^15–17^. Furthermore, accessing large geochemical datasets of archaeological artefacts will lead to more robust and large-scope modelling of prehistoric exchange systems^18–20^. However, the current lack of appropriate global data management platform makes it difficult to access and reference relevant archaeological datasets and often induces duplication of individual endeavors.

**Fig. 1:**
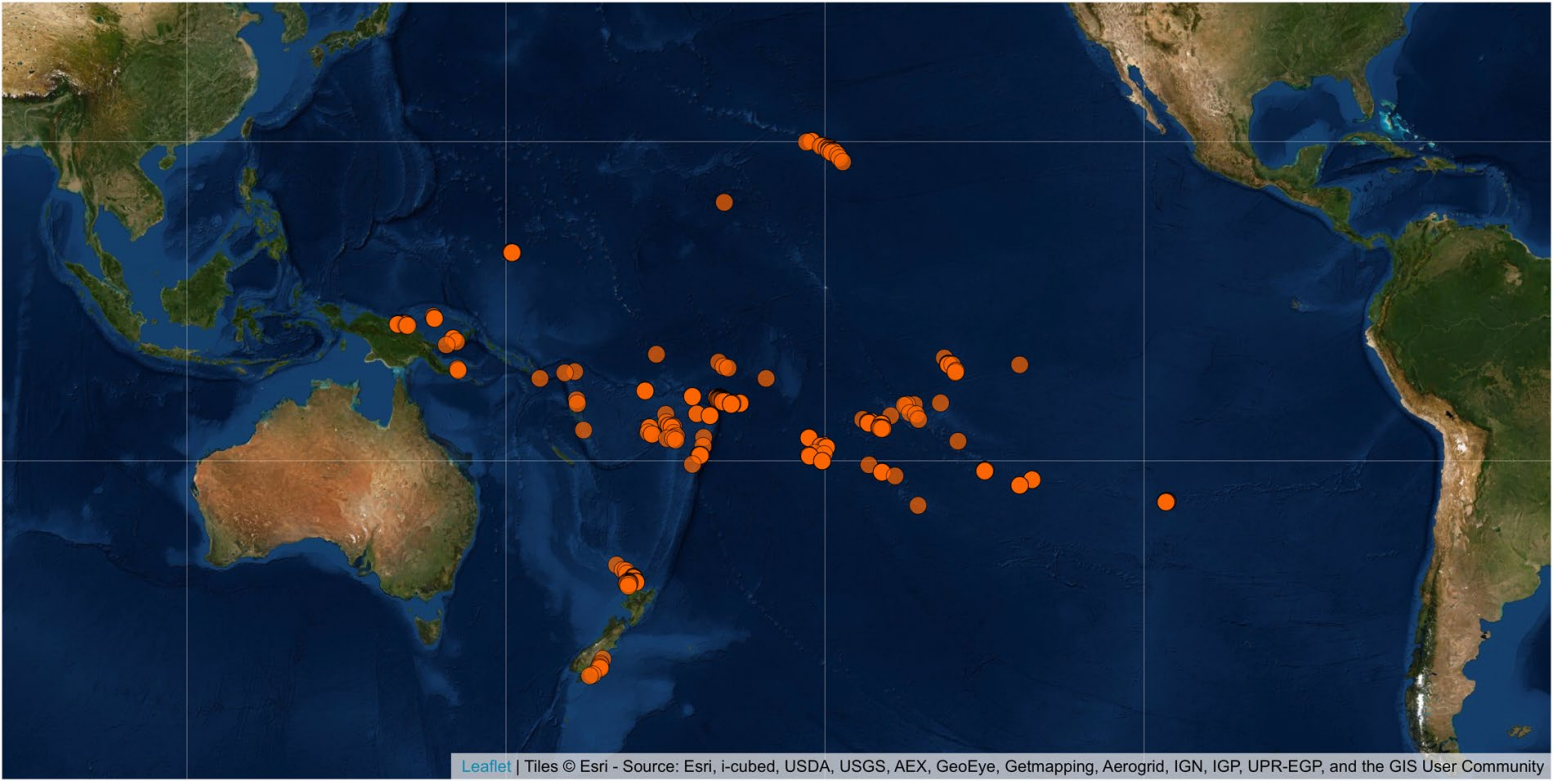
Locations of samples already released in the Pofatu Database.

In this data descriptor, we introduce the Pofatu Database, a curated and open-access database of geochemical data on archaeological materials and sources supported by comprehensive contextual information about individual samples and artefacts, including about the archaeological provenance, and a thorough description of analytical procedures. The goals of the database are (i) to provide easy access to published compositional data of archaeological sources and artefacts, (ii) to assemble contextual archaeological information for each individual sample, (iii) to facilitate reuse of existing data and encourage the appropriate crediting of original data sources, and (iv) to ensure reproducibility and comparability by documenting instrumental details, analytical procedures and reference materials used for calibration purposes or quality control. We provide compositional data as well as contextual metadata for 7759 individual samples with a current focus on archaeological sites across the Pacific Islands (Fig. 1). Our vision is an inclusive and collaborative data resource to activate an operational framework for data sharing in archaeometry, that will progressively include more datasets, and initiate a more global project similar to other online repositories for geological materials already available through a wide geoinformatics network^21–24^. Furthermore, by using common non-proprietary file formats (CSV) and an open source system for storage and version control (Git and GitHub repository), the Pofatu Database provides an analysis-friendly environment that enables transparency and built-in reproducibility of analytical tasks^25^.

## Methods

**Fig. 2:**
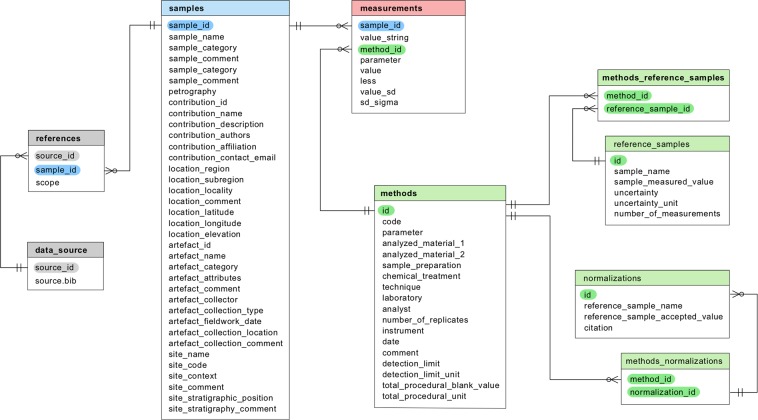
Structure of the Pofatu Database.

The data can be accessed and downloaded from the Zenodo archive (10.5281/zenodo.3670127) and browsed in the Pofatu web application (https://pofatu.clld.org/). The database was designed to contain geochemical compositional data and extensive contextual metadata (sample identification, archaeological provenance, analytical methods, and related bibliographical references), which we compiled to ensure further reuse and reinterpretation of previous provenance analyses (Fig. 2).

The compositional data contains all analytical values for major oxide and trace element compositions, radiogenic and stable isotope ratios, and geochronology. Sample metadata involves the creation of unique identifiers, and a description of sample condition and preparation. Archaeological metadata provides information on the geographical, cultural and stratigraphic context of the parent artefacts (name, category and attributes), the collection origin (collector, date and nature of field research, storage location), and a description of the site and stratigraphic context (name, code, context, stratigraphic position). The reference metadata lists all bibliographical sources of the data and metadata information^26–173^. Methodological metadata ensure a control on data quality and include information about the preparation of samples analytical procedure (technique, laboratory, analyst) as well as the accuracy and reproducibility of published analyses (errors, precision, standard values, correction procedures).

### Data acquisition

All data and metadata in the Pofatu Database and included in this data descriptor release are linked with published resources. Geochemical datasets are extracted from peer-reviewed material, while contextual metadata include information gathered from peer-reviewed articles, monographs, book chapters, and publicly available institutional reports. Original sources are coded in the repository and available as a BibTeX database file, suitable for importing into reference management software. Geochemical datasets are associated with a method identifier, which is unique and defined based on the set of available methodological metadata for a specific set of values.

The process of data acquisition includes:

*Data submission:* Data and metadata are gathered and stored in normalized tables linked by foreign keys. These interrelated tables each contain sets of information on (i) Data source, (ii) Sample and archaeological provenance, (iii) Compositional data, (iv) Primary analytical and method-specific metadata. The Pofatu Database is frequently curated and updated on a regular basis. New datasets and complementary information on previously documented datasets can be submitted using the Data Submission Template and Guidelines available online (https://pofatu.clld.org/about).

*Data validation:* The content of each table is handled manually but several fields are constrained by ontologies, which are built-in form validation in the submission template. Data is also validated using functionality implemented in the Python package pypofatu, which imposes suitable constraints on data like geographic coordinates.

*Data output*: The manually curated “raw” data undergoes an automated processing workflow (implemented in the Python package pypofatu) to create output formats ready for distribution.

For long-term accessibility, the data is converted to a set of interrelated CSV files, described by metadata encoded as JSON-LD (cf. https://www.w3.org/TR/json-ld/, accessed January 30, 2020), following the World Wide Web Consortium (W3C) recommendations^174,175^. Because the compiled data is exclusively made of line-based text files (in CSV format), it is well-suited for long-term access since it has the lowest requirements on processing software, and provides for a transparent history of changes with the version control software Git (cf. https://git-scm.com/, accessed January 30, 2020).

## Data Records

A release of the Pofatu Database is available from the Zenodo archive^176^. Details of the parameters and measurements reported in the database are summarized in Online-only Table 1. Unique identifiers for samples, artefacts and analytical methods were created for each data record, and used as primary and foreign keys to define relationships between tables.

## Technical Validation

Quality control of data and editorial procedures include:

*Data review:* Database contributors who submit a new dataset are asked to be the editor of that specific dataset and to engage in a review of potential missing or inaccurate data. The content of new datasets is systematically cross-checked with the content of original sources and with potentially related content. Authors are contacted when information is missing or when clarifications are needed.

*Duplicate detection:* Since Pofatu assigns semantic, unique identifiers to the objects in the database, and links data from additional tables using these keys (following the recommendations by Wilson and colleagues^177^), data consistency can be checked automatically, e.g. detecting multiple conflicting measurements of the same parameter in the same analysis, or conflicting sample metadata.

*Users feedback:* Data and metadata issues can be reported to pofatu@shh.mpg.de. Editors will be contacted if an issue with one of their datasets is reported.

## Usage Notes

The Pofatu Database provides an analysis-friendly environment^178^ that enables transparency and built-in reproducibility of analytical tasks that can be achieved through freely available softwares or web browsers^25^.

Since the metadata provided with the csv-formatted data files has information about data types as well as relations between the tables making up the dataset, it is automatically loaded into an SQLite database (cf. https://sqlite.org/appfileformat.html, accessed January 29, 2020) for the convenience of the users. This SQLite database is contained in a single file document that can be queried with a high-level query language, has accessible content, is cross-platform, performant, and can be used with multiple programming languages.

The Python package pypofatu used for curating the dataset also provides functionality (built-in SQLite driver) that enables access and queries of the data with Python programs or the pypofatu API, and facilitates running SQL queries against the SQLite database.

Complex queries can be created in various ways and with different computing environments:

- using SQL command line
- using SQL browsers such as SQLite manager or SQLite reader
- using R, with SQL codes in a notebook or packages such as sqldf or dplyr^179,180^
- using the Datasette tool^181^

Data usage instructions are provided in the GitHub repository where the dataset is curated (cf. https://github.com/pofatu/pofatu-data, accessed February 6, 2020). A “cookbook” collects shareable pieces of code and how-to instructions to query the relational database (cf. https://github.com/pofatu/pofatu-data/blob/master/doc/cookbook.md, accessed February 6, 2020), and users are invited to contribute with the “recipes” they used for “cooking” with Pofatu.

## Data Availability

The pypofatu Python package is open-source software, maintained on GitHub and distributed via the Python Package Index (https://pypi.org/project/pypofatu), with released versions archived with Zenodo^182^. The two output formats listed above are created and stored as part of the GitHub repository where the dataset is curated (https://github.com/pofatu/pofatu-data/releases/tag/v1.0.0), and each release of the dataset is also archived on Zenodo^176^. Additionally, the dataset is loaded into a clld^183^ web application, providing an online, browsable user interface for “window-shopping”, before downloading and using the dataset locally. Released versions of the Pofatu dataset meet the requirements on FAIR data as laid out by Wilkinson and colleagues^177^. The data is findable thanks to Zenodo’s integration in the research data landscape on the web, and the metadata we provide. It is accessible via the DOI doled out by Zenodo. “It is interoperable due to the open standards” used to encode the data and reusable because it is provided under an open CC-BY license.

## Online-only Table

**Online-only Table 1:** Overview of fields and content of the Pofatu database.

Table	Field name	Field content^a^	Format^a,b,c,d^
sources	ID	Unique identifier of the bibliographic reference	ID(1)
	Entry_Type	Zotero item types and fields	Zotero item types and fields
	annotation		
	author		
	bookauthor		
	booktitle		
	date		
	doi		
	edition		
	editor		
	editora		
	editoratype		
	institution		
	isbn		
	issn		
	issue		
	journaltitle		
	keywords		
	langid		
	location		
	note		
	number		
	pages		
	pagetotal		
	pmcid		
	pmid		
	publisher		
	rights		
	series		
	shortjournal		
	shorttitle		
	title		
	type		
	url		
	urldate		
	volume		
	volumes		
samples	ID	Unique identifier of the analyzed sample	ID(1)
	sample_name	Published name of the sample	CHAR
	sample_category	Status of the analyzed sample: source or artefact	CHAR(CV)
	sample_comment	Supplementary information about the status of the sample. Eg. “Artefact used as a source”	CHAR
	petrography	Petrographic identification of the analyzed material	CHAR
	contribution_id	Unique identifier of the dataset	CHAR
	contribution_name	Descriptive title of the dataset	CHAR
	contribution_description	Brief description (abstract) of dataset	CHAR
	contribution_authors	Author(s) of the dataset (Last, First name)	CHAR
	contribution_affiliation	Institution(s) of the author(s)	CHAR
	contribution_contact_email	Contact email for the creator of the template	CHAR
	location_region	Name of the region where the parent artefact was collected	CHAR
	location_subregion	Name of the sub-region where the parent artefact was collected	CHAR
	location_locality	Name of the locality where the parent artefact was collected	CHAR
	location_comment	Supplementary information about the provenance of the parent artefact	CHAR
	location_latitude	Coordinates in decimal degrees (negative indicate South)	NUM
	location_longitude	Coordinates in decimal degrees (negative indicate West)	NUM
	location_elevation	Elevation in meters, with respect to sea level	NUM
	artefact_id	Unique identifier for the parent artefact of the analyzed sample	ID(1)
	artefact_name	Name of the parent artefact in the original publication	CHAR
	artefact_category	General artefact classification	CHAR(CV)
	artefact_attributes	General attributes (fragmentation, raw material, etc.)	CHAR(CV)
	artefact_comment	Supplementary information about the parent artefact	CHAR
	artefact_collector	Principal investigator of fieldwork collection	CHAR
	artefact_collection_type	Type of field research: excavation or survey	CHAR(CV)
	artefact_fieldwork_date	Period/dates of the field research	CHAR
	artefact_collection_location	Name of the institution managing the collection	CHAR
	artefact_collection_comment	Supplementary information about the archaeological collection	CHAR
	site_name	Name given to the archaeological site in the literature	CHAR
	site_code	Code of the archaeological site in the literature	CHAR
	site_context	Assumed function of the archaeological site based on the literature	CHAR(CV)
	site_comment	Supplementary information about the archaeological site	CHAR
	site_stratigraphic_position	Original stratigraphic context of the parent artefact	CHAR
	site_stratigraphy_comment	Supplementary information about the site stratigraphy	CHAR
measurements	Sample_ID	Unique identifier of the analyzed sample	ID(2)
	value_string	String value of: oxides (wt%), trace elements and REE (ppm), radiogenic isotopes, geochronology	NUM
	Method_ID	Unique identifier of the analytical method	ID(2)
	parameter	Geochemical parameter analyzed	NUM
	value	Measured value of: oxides (wt%), trace elements and REE (ppm), radiogenic isotopes, geochronology	NUM
	less	Value_string actually inferior to expressed value: yes/no	CHAR(CV)
	value_sd	Standard deviation (error) value	NUM
	sd_sigma	Standard deviation confidence interval: 1σ or 2σ	CHAR(CV)
methods	ID	Unique identifier of the analytical method for each parameter of a give dataset	ID(1)
	code	Identifier of the analytical method for a given dataset	ID(1)
	parameter	Geochemical parameter analyzed (fields of compositional data)	CHAR(CV)
	analyzed_material_1	Description of the analyzed material: whole rock, volcanic glass, mineral	CHAR(CV)
	analyzed_material_2	Description of the analyzed sample: core sample, fused disks, sample surface, powder	CHAR(CV)
	sample_preparation	Description of the sample preparation	CHAR
	chemical_treatment	Description of the sample chemical treatment	CHAR
	technique	Analytical technique	CHAR(CV)
	laboratory	Name of the institution of the analysis	CHAR
	analyst	Name of the analyst	CHAR
	number_of_replicates	Number of replicates	CHAR
	instrument	Instrument type and name	CHAR
	date	Period/dates of the analysis	CHAR
	comment	Supplementary information about the analysis. e.g. reference to the method description in the literature	CHAR
	detection_limit	Lower limit of detection	NUM
	detection_limit_unit	Detection limit unit	CHAR
	total_procedural_blank_value	Blank value	NUM
	total_procedural_unit	Blank value unit	CHAR
methods_reference_samples	Method_ID	Unique identifier of the analytical method	ID(2)
	Reference_sample_ID	Reference sample name (international standard)	ID(1)
reference_samples	ID	Reference sample name (international standard)	ID(2)
	sample_name	Name of international standard in literature	CHAR
	sample_measured_value	Reference measurement value	NUM
	uncertainty	Reference standard deviation	NUM
	uncertainty_unit	Reference uncertainty unit: %, ppm, 1σ or 2σ	CHAR
	number_of_measurements	Number of measurements	NUM
methods_normalizations	Method_ID	Unique identifier of the analytical method	ID(2)
	Normalization_ID	Reference sample name used for normalization	ID(1)
normalizations	ID	Reference sample name used for normalization	ID(2)
	reference_sample_name	Reference sample name used for normalization	CHAR
	reference_sample_accepted_value	Reference measurement accepted value used for normalization	NUM
	citation	Reference of reference sample in the literature	CHAR
references	Source_ID	Unique identifier of the bibliographic reference	ID(2)
	Sample_ID	Unique identifier of the analyzed sample	ID(2)
	scope	Scope of sample information documented by reference: sample, artefact or site	CHAR(CV)

^a^see Zotero item types and fields at: https://www.zotero.org/support/kb/item_types_and_fields.

^b^CHAR, character; NUM, number.

^c^CV, controlled vocabulary.

^d^ID(1), primary key; ID(2), foreign key.
